# Supplementary material for: Combination RSL3 Treatment Sensitizes Ferroptosis- and EGFR-Inhibition-Resistant HNSCCs to Cetuximab
Source: Int J Mol Sci. 2022 Aug 12;23(16):9014. doi: 10.3390/ijms23169014 (PMC9409433; doi:10.3390/ijms23169014)
Supplement: Supplementary file 1 [file ijms-23-09014-s001.zip › ijms-1823627-supplementary.pdf]

Liu et al., Figure S1 Sequencing result of RAS genes in HNSCC cells

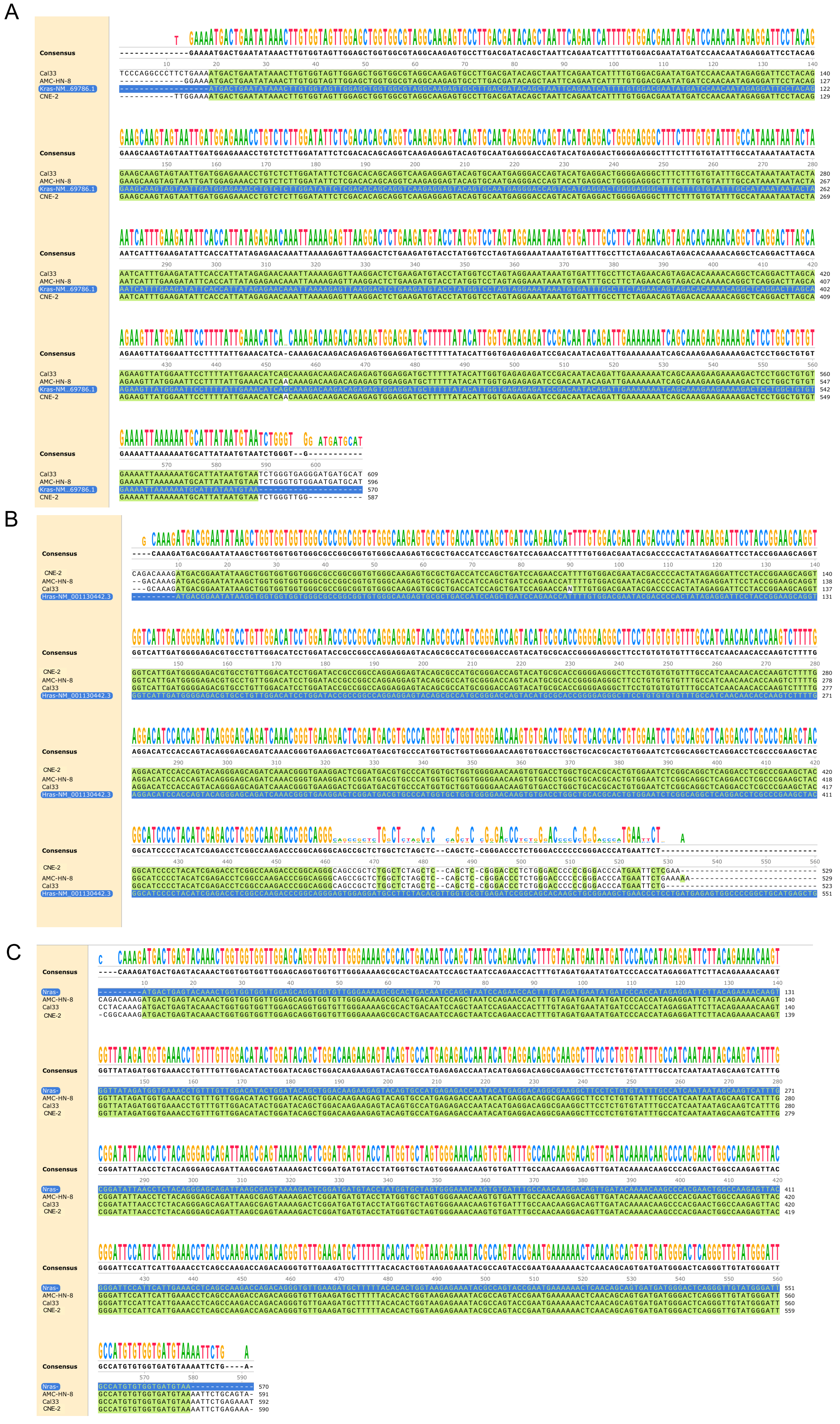

Supplement Table S1 Primers' sequences

Primers	5'→3'
qGAPDH-F	GTCTCCTCTGACTTCAACAGCG
qGAPDH-R	ACCACCCTGTTGCTGTAGCCAA
qKRAS-F	TGTGGACGAATATGATCCAACA
qKRAS-R	GCAAATACACAAAGAAAGCCCT
qNRAS-F	GAAACCTCAGCCAAGACCAGAC
qNRAS-R	GGCAATCCCATACAACCCTGAG
qHRAS-F	ACGCACTGTGGAATCTCGGCAG
qHRAS-R	TCACGCACCAACGTGTAGAAGG
qTF-F	GTACTCCAAGTTTCTGATGCAC
qTF-R	CTCTTGATGACAAACTGGATGC
qTFRC-F	ATCGGTTGGTGCCACTGAATGG
qTFRC-R	ACAACAGTGGGCTGGCAGAAAC
qAURKA-F	GCAACCAGTGTACCTCATCCTG
qAURKA-R	AAGTCTTCCAAAGCCCACTGCC
qFTH-F	TGAAGCTGCAGAACCAACGAGG
qFTH-R	GCACACTCCATTGCATTCAGCC
qEGFR-F	AACACCCTGGTCTGGAAGTACG
qEGFR-R	TCGTTGGACAGCCTTCAAGACC
